# HDAC1在肺癌中的潜在作用机制及研究进展

**DOI:** 10.3779/j.issn.1009-3419.2026.106.11

**Published:** 2026-04-20

**Authors:** Mingzhu LIU, Chuanlin WANG, Jin HU, Guangqiang ZHAO, Yunchao HUANG, Lan ZHOU

**Affiliations:** ^1^650504 昆明，昆明医科大学第三附属医院，云南省肿瘤医院，北京大学肿瘤医院云南医院临床营养科; ^1^Department of Clinical Nutritiony, The Third Affiliated Hospital of Kunming Medical University, Yunnan Cancer Hospital, Peking University Cancer Hospital Yunnan, Kunming 650504, China; ^2^650504 昆明，昆明医科大学第三附属医院，云南省肿瘤医院，北京大学肿瘤医院云南医院，胸外科; ^2^Department of Chest Surgery, The Third Affiliated Hospital of Kunming Medical University, Yunnan Cancer Hospital, Peking University Cancer Hospital Yunnan, Kunming 650504, China

**Keywords:** 肺肿瘤, HDAC1, 表观遗传修饰, HDAC1抑制剂, Lung neoplasms, HDAC1, Epigenetic modifications, HDAC1 inhibitors

## Abstract

肺癌是全球发病率与死亡率最高的恶性肿瘤。当前治疗手段疗效有限，患者预后不佳，亟需探索新的治疗靶点与策略。表观遗传修饰失调与肿瘤发生发展密切相关，其中组蛋白去乙酰化酶1（histone deacetylase 1, HDAC1）作为关键的表观遗传调控分子，参与调控细胞增殖、分化、凋亡等生物学过程。HDAC1在肺癌中呈高表达，且其表达水平与肺癌恶性程度、临床分期及不良预后密切关联。HDAC1异常激活也是肺癌化疗、靶向治疗及免疫治疗耐药的核心因素之一。HDAC1抑制剂的研发为肺癌治疗提供了新方向，且与化疗、靶向治疗及免疫治疗联用能够显著增强协同抗肿瘤效果。本文系统综述了HDAC1的结构特征与生理功能，深入探讨其在肺癌中的调控机制，阐述其与肺癌耐药的关联，并总结HDAC1抑制剂的研发进展、临床试验现状及面临的挑战，以期为肺癌的精准治疗提供新思路。

## Abstract (en)

Lung cancer is the malignant tumor with the highest incidence and mortality rate worldwide. The current treatment methods have limited efficacy, and the prognosis of patients is poor. There is an urgent need to explore new therapeutic targets and strategies. Epigenetic modification disorders are closely related to the occurrence and development of tumors. Among them, histone deacetylase 1 (HDAC1), as a key epigenetic regulatory molecule, participates in the regulation of biological processes such as cell proliferation, differentiation, and apoptosis. HDAC1 is highly expressed in lung cancer, and its expression level is closely related to the malignancy degree, clinical stage, and poor prognosis of lung cancer. Abnormal activation of HDAC1 is also one of the core factors for the resistance of lung cancer to chemotherapy, targeted therapy, and immunotherapy. The development of HDAC1 inhibitors provides a new direction for the treatment of lung cancer, and the combination with chemotherapy, targeted therapy, and immunotherapy can significantly enhance the synergistic anti-tumor effect. This article systematically reviews the structural characteristics and physiological functions of HDAC1, deeply explores its regulatory mechanism in lung cancer, elaborates on its association with lung cancer resistance, and summarizes the research and development progress, clinical trial status, and challenges of HDAC1 inhibitors, with the aim of providing new ideas for the precise treatment of lung cancer.

肺癌是全球发病率和死亡率最高的恶性肿瘤，死亡病例达181万，占癌症总死亡人数的18.7%^[1]^。尽管手术、化疗、靶向治疗及免疫治疗等手段不断发展，但肺癌患者的生存率仍不理想^[2]^。因此，需要探寻更有效的方法来改善肺癌的治疗和预后。

表观遗传调控的改变会导致基因表达异常，而基因表达异常往往与肿瘤的发生发展密切相关^[3]^。表观遗传修饰是指在不改变DNA序列的情况下，通过组蛋白修饰、非编码RNA调控等方式影响基因表达，其中组蛋白修饰是关键的表观遗传机制之一^[4-6]^。组蛋白乙酰化状态由组蛋白乙酰转移酶（histone acetyltransferases, HATs）和组蛋白去乙酰化酶（histone deacetylases, HDACs）共同调控。HATs催化组蛋白乙酰化，使染色质松散并促进基因转录；HDACs则去除组蛋白乙酰基，导致染色质浓缩并抑制基因表达^[7]^。这种动态平衡的失衡会导致抑癌基因沉默、癌基因激活，从而驱动肿瘤发生发展^[8,9]^。

在哺乳动物中，HDACs由18种亚型组成，分为四大类：I、II、III和IV类。经典的HDACs（I、II、IV类）需要锌离子（Zn²⁺）作为辅助因子或催化激活剂，而III类HDACs（SIRT1-7）的酶活性依赖于烟碱氨腺嘌呤二核苷酸（nicotinamide adenine dinucleotide, NAD⁺）。I类HDACs（HDAC1, 2, 3, 8）主要定位于细胞核，参与细胞增殖、分化和周期等基础生命过程^[10]^。II类HDACs可分为IIa（HDAC4, 5, 7, 9）和IIb（HDAC6, 10），IIa类HDACs可在细胞核与细胞质之间穿梭，IIb类HDACs主要在细胞质中表达^[11,12]^。III类HDACs（SIRT1-7）依赖于细胞代谢辅因子NAD⁺，能够直接感应细胞的能量与氧化还原状态^[13]^。IV类HDACs仅包含HDAC11，主要定位于细胞核，作为转录调节因子在免疫调节中发挥重要作用^[14]^。HDAC1在多种癌症中高表达且与不良预后有关^[15,16]^。

HDAC1可以通过表观遗传调控影响细胞增殖、凋亡与侵袭转移，并且参与肿瘤免疫微环境塑造及治疗耐药形成^[17]^。近年来，HDAC抑制剂的研发为肺癌治疗提供了新方向。HDAC抑制剂与化疗、靶向治疗及免疫治疗的联合方案展现出协同增效潜力^[18,19]^。本文系统综述了HDAC1抑制剂在肺癌中的作用机制，强调了其作为治疗靶点的潜力，以期对肺癌治疗提供新的治疗思路。

## 1 HDAC1的结构特征与生理功能

HDAC1包含一个核心催化结构域，该结构域依赖Zn²⁺作为辅助因子，通过结合底物蛋白的乙酰化赖氨酸残基，催化乙酰基的去除^[20]^。与II类HDACs不同，HDAC1缺乏核输出信号，仅含核定位信号，因此主要定位于细胞核内，通过与染色质直接结合发挥转录调控作用^[21]^。例如，在细胞周期中HDAC1被招募到周期蛋白依赖激酶抑制因子1A（cyclin-dependent kinase inhibitor 1A, *p21*）基因的启动子区域，通过使组蛋白去乙酰化，形成浓缩的染色质结构，从而沉默*p21*基因的转录^[22]^。HDAC1常与其他蛋白形成多亚基复合物，如SIN3转录调节因子家族成员A（SIN3 transcription regulator family member A, mSin3A）、NuRD复合物等，这些复合物的形成不仅增强了HDAC1的催化活性，还赋予其对特定靶基因的调控特异性^[23-27]^。除组蛋白外，HDAC1也作用于肿瘤蛋白53（tumor protein 53, p53）、成肌分化因子（myogenic differentiation, MyoD）、缺氧诱导因子1α（hypoxia inducible factor 1 subunit alpha, HIF-1α）、DNA甲基转移酶1（DNA methyltransferase 1, DNMT1）等非组蛋白底物，通过去乙酰化调控其稳定性、亚细胞定位或蛋白-蛋白相互作用，进而影响细胞周期检验点、分化程序及应激反应^[28-31]^。Mal等^[29]^研究发现，HDAC1可与MyoD直接结合，并使其保持脱乙酰化状态，去乙酰化的MyoD无法激活下游肌肉特异性基因的转录，从而阻碍肌细胞进入分化程序，影响肌细胞的分化进程。HDAC1下调也可导致p53乙酰化水平升高，促进细胞凋亡^[28]^。

HDAC1对组蛋白修饰与非组蛋白调控的双重功能，协调着细胞的增殖、分化等功能，其功能失调与肿瘤发生发展密切关联。

## 2 HDAC1在肺癌发生发展中的作用机制

临床研究^[32]^表明，HDAC1在肺癌组织及细胞系中呈显著高表达，其表达水平与肺癌的恶性程度及不良预后有密切关联。以下按非小细胞肺癌（non-small cell lung cancer, NSCLC）和小细胞肺癌（small cell lung cancer, SCLC）分别阐述HDAC1的作用机制。

### 2.1 HDAC1在NSCLC发生发展中的作用

#### 2.1.1 HDAC1表达异常与临床意义

HDAC1在NSCLC组织及细胞系中呈显著高表达。陈艳红等^[33]^对117例NSCLC患者的血清样本分析发现，NSCLC患者血清HDAC1浓度显著高于健康体检者。且术前III-IV期患者的HDAC1水平显著高于I-II期患者。在肺鳞状细胞癌中HDAC1高表达与肿瘤大小、肿瘤原发灶-淋巴结-转移（tumor-node-metastasis, TNM）分期有关^[34]^。细胞模型也验证了这一趋势，在A549、NCI-H1299等NSCLC细胞系中，HDAC1的mRNA及蛋白水平均显著高于正常肺上皮细胞^[35]^。这些证据表明，HDAC1的表达异常是NSCLC发生发展的重要影响因素。

#### 2.1.2 促进细胞周期进展与增殖

在NSCLC细胞中，HDAC1通过多种机制驱动细胞恶性增殖：一方面，HDAC1可结合抑癌基因启动子区域，通过组蛋白去乙酰化作用，HDAC1诱导*p21*、磷酸酶与张力蛋白同源物基因（phosphatase and tensin homolog, *PTEN*）等抑癌基因位点的染色质浓缩，造成转录沉默，进而导致细胞周期检查点失效，促进细胞异常增殖（图1）^[31,36]^；另一方面，HDAC1能够激活细胞周期相关通路，推动肺癌细胞周期进程。在肺鳞状细胞癌中，HDAC1可对视网膜母细胞瘤蛋白（retinoblastoma protein, Rb）进行去乙酰化修饰，促进Rb蛋白磷酸化（图1）。这解除了Rb对E2启动子结合因子（E2 promoter binding factor, E2F）转录因子的抑制作用，进而激活G_1_/S-特异性周期蛋白D1（G_1_/S-specific cyclin-D1, Cyclin D1）、细胞周期蛋白E（Cyclin E）等细胞周期蛋白的表达，推动细胞从G_1_期进入S期^[34]^。此外，HDAC1还可与HDAC2协同，通过HRP-3被招募至E2F1启动子区。通过组蛋白去乙酰化抑制E2F1转录，进一步调控G_1_/S期转换进程^[37]^。作为转录共抑制子，HDAC1可与GSE1螺旋蛋白（gse1 coiled-coil protein, GSE1）结合，调控肿瘤抑制基因Kruppel样转录因子6（Kruppel-like factor 6, KLF6）的表达^[38]^（图1）。

**图1:**
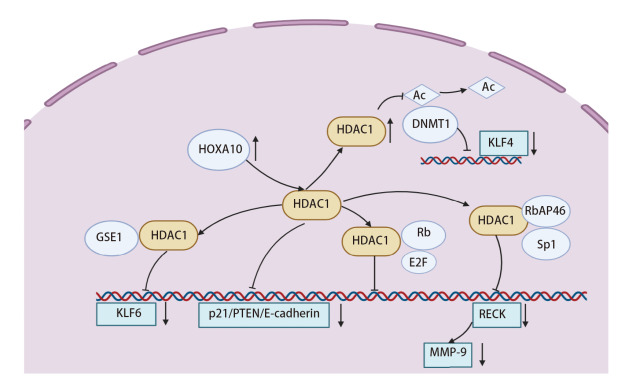
NSCLC中HDAC1部分信号通路图

#### 2.1.3 促进上皮-间质转化（epithelial-mesenchymal transition, EMT）与侵袭转移

HDAC1是NSCLC细胞EMT进程的重要调控因子。在Ras信号通路激活的背景下，HDAC1可被视网膜母细胞瘤相关蛋白46（Rb-associated protein 46, RbAp46）招募，与转录因子Sp1形成转录抑制复合体。该复合体直接抑制转移抑制基因RECK（reversion inducing cysteine rich protein with Kazal motifs）的表达。RECK表达下调会解除其对基质金属蛋白酶9（matrix metallopeptidase 9, MMP-9）的抑制作用，最终增强肺癌细胞的侵袭能力^[39]^（图1）。在肺腺癌中HDAC1可通过去乙酰化修饰增强Snail蛋白的稳定性，促进E-钙黏蛋白（*E-cadherin*）启动子区域染色质浓缩，抑制其转录^[40]^。此外，ZEB1可招募HDAC1/2到*E-cadherin*启动子，抑制H3和H4乙酰化，下调其表达。KLF10通过HDAC1调控*Snail2*表达，参与肺腺癌EMT进程^[41]^。然而，目前关于HDAC1在不同NSCLC亚型中对EMT核心转录因子选择性调控机制仍需更多研究阐明。

#### 2.1.4 与其他表观遗传修饰的相互作用

HDAC1介导的去乙酰化修饰并非孤立存在，而是与其他表观遗传修饰存在广泛的相互作用，共同参与肺癌的发生发展。

DNA甲基转移酶（DNA methyltransferases, DNMTs）催化的启动子区高甲基化通常与基因沉默相关。Ma等^[31]^研究发现，在肺腺癌中HOXA10可通过促进HDAC1的表达，进而上调DNMT1的表达。DNMT1催化肿瘤抑制基因*KLF4*启动子区域的DNA甲基化，导致*KLF4*基因沉默，最终促进肺腺癌发展（图1）。这表明HDAC1介导的组蛋白去乙酰化与DNMT1介导的DNA甲基化相互协同，共同抑制抑癌基因表达。研究^[42]^发现，在EZH2功能获得性突变型弥漫性大B细胞淋巴瘤中，HDAC1/2与EZH2-PRC2复合物相互作用，促进H3K27去乙酰化，为EZH2催化的H3K27me3创造条件。Takashina等^[43]^发现，在NSCLC中HDAC1与EZH2联合抑制具有协同抗增殖作用，可有效抑制表皮生长因子受体（epidermal growth factor receptor, EGFR）野生型及突变型细胞的生长。

Min等^[44]^发现，RSF1招募HDAC1至DNA损伤位点，介导H2A-K118去乙酰化，进而促进H2A-K119泛素化，调控染色质结构和DNA损伤修复。但在肺癌中HDAC1与组蛋白泛素化之间的相互作用需进一步研究。

以上相互作用机制共同构成了NSCLC中复杂的表观遗传调控网络，但其在肺癌不同发展阶段的动态变化仍有待进一步探索。

### 2.2 HDAC1在SCLC发生发展中的作用

与NSCLC相比，HDAC1在SCLC中的研究相对不足，但现有证据表明其具有独特的作用机制。SCLC的显著分子特征之一是Notch1信号通路的沉默。研究^[45]^表明，组蛋白去乙酰化是SCLC中Notch1表观遗传失活的重要原因。使用HDAC抑制剂（如TSA、VPA）处理SCLC细胞后，Notch1启动子区域乙酰化水平升高，Notch1表达恢复，抑制其增殖和迁移^[45-47]^。这提示HDAC1是维持SCLC神经内分泌表型的关键因子。

HDAC1通过调控免疫相关分子的表达，显著参与肺癌免疫抑制微环境的构建，为肿瘤细胞的免疫逃逸创造条件。c-Myc（myelocytomatosis oncogene）是一种关键的癌基因转录因子，在多种肿瘤中异常激活，参与调控细胞增殖、代谢及免疫应答等过程。赵沛妍等^[48]^研究证实，在Y亚型SCLC细胞中，c-Myc可通过结合HDAC1，抑制其去乙酰化酶活性，进而通过表观遗传机制上调免疫激活配体MICA/B的表达。MICA/B是自然杀伤（natural killer, NK）细胞表面NK组2成员D（NK group 2 member D, NKG2D）受体的配体，其表达升高可增强NK细胞对肿瘤细胞的识别与杀伤能力，而HDAC1的异常激活会削弱这一过程，促进肿瘤免疫逃逸。

## 3 HDAC1参与肺癌治疗耐药

目前关于HDAC1在SCLC中直接介导耐药机制的研究相对有限，本文主要探讨HDAC1在NSCLC中的耐药机制。

### 3.1 化疗耐药

在NSCLC化疗耐药中，HDAC1通过多种机制发挥作用。Lu等^[49]^研究发现，泛素特异性肽酶5（ubiquitin specific peptidase 5, USP5）依赖的HDAC1通过调节Rab相互作用溶酶体蛋白（Rab interacting lysosomal protein, RILP）乙酰化水平，促进NSCLC的恶性进展及顺铂耐药，并通过体外实验证实，敲低HDAC1可显著提高体内肿瘤对顺铂的敏感性。此外，Wang等^[50]^证实，HDAC1可参与形成C3b/SIN3A/HDAC复合物。该复合物增强HDAC1与GADD45A启动子的结合能力，降低组蛋白H3的乙酰化水平，在表观遗传层面抑制GADD45A的表达。过表达GADD45A可促进紫杉醇诱导的细胞凋亡，并增强耐药细胞对PTX的敏感性。而GADD45A表达下调会削弱紫杉醇的细胞毒性，导致耐药发生。

### 3.2 靶向治疗耐药

EGFR-酪氨酸激酶抑制剂（EGFR-tyrosine kinase inhibitors, EGFR-TKIs）是EGFR突变型NSCLC患者的一线治疗药物。但多数患者在治疗过程中会出现耐药现象，HDAC1的异常表达是耐药机制之一。HDAC1可上调细胞间质上皮转换因子（cellular-mesenchymal epithelial transition factor, c-Met），c-Met的异常激活可绕过EGFR信号通路，促进肿瘤细胞增殖与存活，进而介导NSCLC对EGFR-TKIs产生耐药^[51]^。He等^[52]^研究发现，HDAC1抑制剂GCJ-490A与吉非替尼联用可通过抑制HDAC1，实现丝氨酸特异蛋白激酶（IκB kinase alpha, IKKα）表达上调与c-Met表达下调，从而阻断c-Met介导的耐药，恢复吉非替尼对耐药肺癌细胞的抑制活性。此外，使用HDAC抑制剂奎诺司他预处理会下调miR-200c的表达，导致ZEB1表达升高，促进肿瘤细胞对克唑替尼[间变性淋巴瘤激酶（anaplastic lymphoma kinase, ALK）抑制剂]的敏感性^[53]^。

### 3.3 免疫治疗耐药

HDAC1还与肺癌免疫治疗密切关联。抑制HDAC1活性可诱导NKG2D配体的表达。NKG2D配体与NK细胞表面的NKG2D受体结合是激活NK细胞杀伤活性的关键。上调NKG2D配体的表达水平可增强NK细胞对肺癌细胞的杀伤活性，进而增强机体的抗肿瘤免疫应答^[54]^。

## 4 HDAC1在肺癌治疗中的研究进展

### 4.1 HDAC1抑制剂的类型与作用机制

依据HDAC1抑制剂对HDAC亚型的选择性，可分为泛HDAC抑制剂和选择性HDAC1抑制剂。泛HDAC抑制剂（如伏立诺他）通过与HDAC1的催化结构域相结合，竞争性占据其Zn²⁺结合位点，阻断组蛋白去乙酰化反应。该过程可显著上调组蛋白乙酰化水平，激活抑癌基因表达，最终诱导肿瘤细胞周期停滞与凋亡^[55,56]^。选择性抑制剂则通过优化分子结构，增强对HDAC1的特异性结合能力，减少对其他HDAC亚型的抑制作用，以降低药物副作用。例如，罗米地辛（Romidepsin, FK228）作为一种选择性HDAC1和HDAC2抑制剂，可与HDAC1催化结构域形成共价键，实现对其活性的不可逆抑制^[57]^。罗米地辛可显著上调NKG2D配体的表达水平，增强NK细胞对肺癌细胞的杀伤活性^[54]^。

近年来，蛋白水解靶向嵌合体（proteolysis-targeting chimeras, PROTAC）技术为HDAC1降解提供了新策略。与传统抑制剂不同，PROTAC通过招募E3泛素连接酶诱导靶蛋白泛素化降解，可实现对HDAC1的功能性清除而非仅催化抑制^[58]^。目前，针对HDAC1的PROTAC降解剂尚处于早期研发阶段。已有研究^[59]^构建了基于Cereblon E3连接酶的HDAC1靶向降解分子。然而，HDAC1 PROTAC降解剂在肺癌中的研究仍为空白，其成药性、脱靶效应及体内代谢稳定性有待进一步验证。

### 4.2 肺癌治疗中HDAC1抑制剂的应用

尽管HDAC1抑制剂在临床前研究中显示出显著的肺癌细胞杀伤活性，但单药治疗在肺癌临床试验中的效果有限。研究^[60]^发现，早期II期临床试验的结果中，伏立诺他单药治疗在复发或难治性NSCLC患者中未显示出明显的抗肿瘤活性，联合治疗是克服HDAC1抑制剂单药局限性的关键。

#### 4.2.1 联合化疗

伏立诺他在NSCLC细胞模型中与卡铂、紫杉醇具有良好的协同作用，能够提升这两种化疗药物对NSCLC患者的治疗效果^[61]^。在临床II期试验^[62]^中，伏立诺他联合卡铂/紫杉醇一线治疗晚期NSCLC的客观缓解率（objective response rate, ORR）为34%，高于安慰剂组的12%，但18%的患者发生4级血小板减少（安慰剂组3%）。然而，Panobinostat联合卡铂/依托泊苷I期试验因严重血小板减少和发热性中性粒细胞减少而终止^[63]^。伏立诺他与顺铂联用可松弛肿瘤细胞的染色质结构，提高顺铂作用靶点的可及性，进而增强化疗药物的细胞毒性^[64-66]^。Belinostat联合卡铂/紫杉醇治疗初治晚期NSCLC的I期试验^[67]^显示，ORR为35%，中位无进展生存期（progression-free survival, PFS）为5.7个月，常见不良反应是疲劳、恶心、腹泻和中性粒细胞减少（表1^[62,63,67]^）。

**表1:** NSCLC中HDAC1抑制剂主要临床试验

Drug	Clinical trial phase	Joint plan	Population	Curative effect index	Main adverse reactions
Vorinostat^[62]^	II	Carboplatin+PTX	Advanced NSCLC	ORR: 34% vs 12%	Grade 4 thrombocytopenia (18%)
Belinostat^[67]^	I	Carboplatin+PTX	Advanced NSCLC	ORR: 35%；Median PFS: 5.7 mon	Fatigue, nausea, diarrhea, neutropenia
Panobinostat^[63]^	I	Carboplatin+Etoposide	Advanced NSCLC	Terminated	Severe thrombocytopenia, febrile neutropenia

PTX: Paclitaxel; ORR: objective response rate; PFS: progression-free survival.

#### 4.2.2 联合靶向治疗

联合使用HDAC1抑制剂GCJ-490A与吉非替尼，可通过抑制HDAC1来上调IKKα表达、下调c-Met表达，进而阻断c-Met所介导的耐药机制，使吉非替尼重新有效抑制耐药肺癌细胞的生长^[52]^。此外，Lin等^[68]^研究发现，HDAC1抑制诱导的DUSP1表达可介导吉非替尼敏感性，在NSCLC中发挥协同增效作用。

#### 4.2.3 联合免疫治疗

在免疫治疗联合方案中，HDAC1抑制剂能够重塑肿瘤免疫微环境，增强免疫检查点抑制剂的疗效。FK228可通过上调肺癌细胞表面NKG2D配体的表达，增强NK细胞的杀伤活性，同时降低调节性T细胞的肿瘤浸润水平，促进效应T细胞活化^[54]^。此外，HDAC抑制剂与电离辐射联用可协同上调NKG2D配体表达，进一步提升NK细胞对肺癌细胞的杀伤效能^[69]^。

在临床试验方面，伏立诺他联合帕博利珠单抗治疗晚期NSCLC的I/IB期试验^[70]^纳入33例患者，结果显示疾病控制率（disease control rate, DCR）达67%，不良反应主要为疲劳（33%）和恶心/呕吐（27%）。

### 4.3 HDAC1抑制剂研发的挑战与展望

HDAC1抑制剂在肺癌治疗中展现出较大潜力，但其临床转化仍面临诸多挑战。泛HDAC抑制剂在抑制HDAC1的同时，会非特异性地作用于其他HDAC亚型，进而引发疲倦、恶心、呕吐、血小板减少及中性粒细胞减少等不良反应，增加临床用药风险^[71]^。此外，HDAC1与HDAC2的氨基酸序列高度同源，导致开发选择性抑制HDAC1而不影响HDAC2的抑制剂极为困难。

现有HDAC1抑制剂在体内代谢较快且肿瘤组织靶向性不足，导致有效药物浓度难以维持，递送效率低；同时，不同肺癌亚型及患者个体间HDAC1的表达水平与下游调控网络存在显著差异，这种异质性可能导致疗效不稳定，因此开发可靠的HDAC1活性或表达水平生物标志物以筛选优势人群至关重要；此外，肿瘤细胞可通过上调其他HDAC亚型表达、增强药物外排等机制产生耐药性^[72]^。联合用药和新型药物开发虽是克服耐药的关键策略，但其最佳方案及用药时序仍需大规模临床试验验证。

## 5 总结

HDAC1作为表观遗传调控的关键分子，在肺癌的发生发展中发挥多重作用，通过抑制抑癌基因、调控细胞周期促进肺癌细胞增殖，HDAC1的异常激活也是耐药的重要驱动因素。HDAC1抑制剂在肺癌联合治疗中展现出显著潜力，在临床前与早期临床试验中取得积极结果。然而，HDAC1在肺癌中的调控机制仍存在诸多未知，需进一步探索。未来，随着高选择性抑制剂和新型降解剂的开发及联合治疗策略的完善，HDAC1靶向治疗有望成为肺癌精准治疗的重要组成部分，为改善患者预后提供新的选择。
